# IGSF1 Deficiency: Lessons From an Extensive Case Series and Recommendations for Clinical Management

**DOI:** 10.1210/jc.2015-3880

**Published:** 2016-02-03

**Authors:** S. D. Joustra, C. A. Heinen, N. Schoenmakers, M. Bonomi, B. E. P. B. Ballieux, M.-O. Turgeon, D. J. Bernard, E. Fliers, A. S. P. van Trotsenburg, M. Losekoot, L. Persani, J. M. Wit, N. R. Biermasz, A. M. Pereira, W. Oostdijk

**Affiliations:** Department of Pediatrics (S.D.J., J.M.W., W.O.), Department of Medicine (S.D.J., N.R.B., A.M.P.), Division of Endocrinology, Department of Clinical Chemistry and Laboratory Medicine (B.E.P.B.), and Department of Clinical Genetics (M.L.), Leiden University Medical Center, 2300 C Leiden, The Netherlands; Department of Pediatric Endocrinology (C.A.H., A.S.P.v.T.), Emma Children's Hospital, and Department of Endocrinology and Metabolism (C.A.H., E.F.), Academic Medical Center, University of Amsterdam, 1100 DE, The Netherlands; University of Cambridge Metabolic Research Laboratories (N.S.), Wellcome Trust-Medical Research Council Institute of Metabolic Science, Addenbrooke's Hospital, Cambridge DB2 2OO, United Kingdom; Division of Endocrine and Metabolic Disorders (M.B.), Instituto di Ricovero e Cura a Carettere Scientifico, Instituto Auxologica Italiano, 20132 Milan, Italy; Department of Clinical Sciences and Community Health (M.B., L.P.), Università degli Studi di Milano, 20122 Milan, Italy; Department of Pharmacology and Therapeutics (M.-O.T., D.J.B.), McGill University, Montréal, Québec, Canada H9X 3V9

## Abstract

**Context:**

Mutations in the immunoglobulin superfamily, member 1 (*IGSF1*) gene cause the X-linked IGSF1 deficiency syndrome consisting of central hypothyroidism, delayed pubertal testosterone rise, adult macroorchidism, variable prolactin deficiency, and occasionally transient partial GH deficiency. Since our first reports, we discovered 20 new families with 18 new pathogenic *IGSF1* mutations.

**Objective:**

We aimed to share data on the largest cohort of patients with IGSF1 deficiency to date and formulate recommendations for clinical management.

**Methods:**

We collected clinical and biochemical characteristics of 69 male patients (35 children, 34 adults) and 56 female *IGSF1* mutation carriers (three children, 53 adults) from 30 unrelated families according to a standardized clinical protocol. At evaluation, boys were treated with levothyroxine in 89%, adult males in 44%, and females in 5% of cases.

**Results:**

Additional symptoms in male patients included small thyroid gland volume (74%), high birth weight (25%), and large head circumference (20%). In general, the timing of pubertal testicular growth was normal or even premature, in contrast to a late rise in T levels. Late adrenarche was observed in patients with prolactin deficiency, and adult dehydroepiandrosterone concentrations were decreased in 40%. Hypocortisolism was observed in 6 of 28 evaluated newborns, although cortisol concentrations were normal later. Waist circumference of male patients was increased in 60%, but blood lipids were normal. Female carriers showed low free T_4_ (FT_4_) and low-normal FT_4_ in 18% and 60%, respectively, delayed age at menarche in 31%, mild prolactin deficiency in 22%, increased waist circumference in 57%, and a negative correlation between FT_4_ concentrations and metabolic parameters.

**Conclusion:**

IGSF1 deficiency represents the most common genetic cause of central hypothyroidism and is associated with multiple other characteristics. Based on these results, we provide recommendations for mutational analysis, endocrine work-up, and long-term care.

## Abstract (precis)

Clinical and biochemical characteristics of 69 male patients and 56 female IGSF1 mutation carriers were collected, providing recommendations for mutational analysis, endocrine work-up, and long-term care.

We previously reported that loss-of-function mutations in the immunoglobulin superfamily, member 1 (*IGSF1*) gene in 11 families causes the X-linked IGSF1 deficiency syndrome (1). In males the phenotype is characterized by congenital central hypothyroidism, delayed testosterone rise in puberty but normal timing of testicular enlargement, adult macroorchidism, partial GH deficiency (GHD) in some patients during childhood (but with high-normal IGF-1 concentrations in adulthood) or lifelong prolactin deficiency, and overweight habitus (1–5). A minority of female heterozygous carriers exhibits central hypothyroidism (2).

*IGSF1* encodes a plasma membrane glycoprotein, and all described *IGSF1* mutations reported to date impair proper glycosylation and trafficking of the protein to the cell surface (1). Furthermore, *Igsf1*-deficient male mice show reduced serum TSH and T_3_ concentrations, decreased pituitary *Trhr* mRNA levels, and larger body size and weight than wild-type mice (1). Yet IGSF1's specific local function remains enigmatic.

Within 3 years after the discovery of this syndrome, we have identified 20 new families carrying 18 new mutations. To provide a more precise description of the clinical characteristics of IGSF1 deficiency, a total of 69 male patients and 56 female carriers were invited for clinical characterization according to a defined uniform protocol. This allowed us to formulate recommendations for the clinical management of these patients.

## Subjects and Methods

### Design

In this descriptive case series, we present data on the clinical and biochemical characteristics of pediatric and adult hemizygous male patients with a mutation in *IGSF1* and of adult female heterozygous carriers. Data were collected according to a clinical protocol approved by the Medical Ethics Committee of the Leiden University Medical Center. All subjects gave written informed consent.

### Subjects

All patients in whom our group identified a pathogenic mutation in *IGSF1* were invited for additional investigations. We collected data on 35 boys (mean age 9.8 y, range 0.2–17.6 y), 34 male adults (48 years, 18–88 y), 3 girls (9.9, 12.2, and 16.4 y), and 53 female adults (48 y, 21–81 y) from 30 unrelated families carrying *IGSF1* mutations. The families were from The Netherlands (n = 16), the United Kingdom (n = 3), Italy (n = 4), Morocco (n = 2), and Argentina, Belgium, Canada, Israel, and the United States (n = 1 each). Some characteristics of 24 males and 18 females from 10 families within this cohort were previously reported (2) but were supplemented with additional data. Fifteen of 34 male adults were treated with levothyroxine at current evaluation, as were 31 of 35 male children, and 3 of 56 females. All others either chose not to be treated with levothyroxine in the absence of complaints or had not yet started treatment after the detection of hypothyroidism in the course of our analysis of family members of probands. Pathogenicity of the variants was determined based on the presence of central hypothyroidism with proper phenotype-genotype segregation, and in silico and in vitro analysis of the mutated proteins, as previously described (1). For four variants, in vitro results appeared normal (see Supplemental Table 1 for a detailed description of the phenotypes of these variants as well as for those of previously published cases).

### Parameters

Body fat percentage was evaluated with bioelectrical impedance analysis, using the Bodystat 1500MDD (Bodystat Limited). Testicular and thyroid gland size was measured in males using standard ultrasonographic imaging. Descriptions of age-specific reference intervals of anthropomorphic measurements, laboratory assays and reference intervals, and definition of hormone deficiencies are shown in the Supplemental Methods.

### Statistics

Statistical analyses are described in the Supplemental Methods.

## Results

### Hypothalamic-pituitary-thyroid axis

All male patients showed central hypothyroidism (Supplemental Figure 1A) and 59% were prolactin deficient (Table 1). The TSH peak 20 minutes after TRH stimulation was decreased in most neonates and in the lower half of the reference range in most other cases (Supplemental Figure 2). Many patients had a small thyroid gland; the volume was less than the 2.5th percentile (7.7 mL) in 74% of cases and unmeasurably small in two patients. Adult thyroid size (n = 17) was smaller in patients on long-term levothyroxine replacement (median 5.6 mL [interquartile range 1.4–6.7 mL], n = 11) than in untreated patients (7.4 mL [interquartile range 6.4–14.8 mL], n = 6) (*P* = .021). The two patients with unmeasurable thyroid size were 68 and 88 years old and started treatment with levothyroxine at the age of 6 years and 30 years, respectively.

**Table 1.** Basic Clinical Characteristics of the Male Study Population

	n	Children	n	Adults
Gestational age at birth, wk	26	40.6 (40.0–41.6)	15	41.0 (39.0–43.0)
Birth weight (SDS)	31	0.9 (0.2–1.7)^a^	21	1.2 (0.7–2.4)^a^
Head circumference (SDS)	26	0.9 (0.5–1.2)^a^	25	1.2 (0.8–2.1)^a^
Height (SDS)	21	−0.6 (−1.4 to −0.2)^a,b^	30	−0.2 (−1.1 to 0.3)
Thyroid volume <P50/<P2.5	21	95%/67%	17	88%/82%
TSH at diagnosis, mU/L^c^	32	2.15 (1.53–3.28)	28	1.70 (1.20–2.76)
FT_4_ at diagnosis, pmol/L^d^	32	9.1 (8.1–9.6)^a^	28	9.0 (8.5–9.4)^a^
Central hypothyroidism	35	100%	34	100%
Prolactin deficiency	34	62%	31	55%
(transient) GH deficiency	35	11%	34	15%^e^
IGF-1 (SDS)^f^	28	−0.6 (−1.0 to 0.4)	30	1.1 (0.2–1.7)^a^
Cortisol, μmol/L^g^	32	0.268 (0.213–0.347)	26	0.381 (0.317–0.464)
Adult testis volume (SDS)			23	3.5 (2.4–5.1)^a^
Adult thyroid volume, mL^h^			17	6.5 (4.6–7.1)^a^

Data are presented as median (interquartile range).

a Different from population median at *P* < .05.

b Only Dutch children.

c TSH normal range: 0.30–4.80 mU/L.

d Normal ranges vary with age and assay. See Supplemental Figure 1 for FT_4_ at diagnosis relative to reference range.

e In these adults, GHD had been transiently present only during childhood and adolescence, except for one patient aged 19 years.

f In patients not treated with rhGH.

g Early-morning fasting withdrawal. In-house reference values for cortisol are 0.100–0.600 μmol/L.

h Reference values in adult men: 19.1 mL (P50) and 7.7 mL (P2.5) (40).

Serum SHBG, an indicator of hypothyroidism in the liver, was significantly correlated with free T_4_ (FT_4_) concentrations in treated and untreated adult patients (*r* = 0.583, *P* = .002) and also after correction for body mass index (BMI) (corrected *P* = .003).

### Growth and development

Gestational age was normal, but birth weight was 0 or greater SD scores (SDS) in 83% and 2.0 or greater SDS in 25% of cases (Table 1). Psychomotor development was generally normal, although four received physical therapy for problems with gross motor skills described as clumsiness. Patients with sufficient available data showed a consistent, typical growth and pubertal development pattern of slow linear growth and delayed bone age during childhood, delayed pubertal T production with subsequent delayed pubertal growth spurt and development of secondary sexual characteristics during adolescence, normal or early timing of the start of testicular growth, and normal adult height, as reported previously (2). Head circumference was slightly increased, being 0 or greater SDS in 89% and 2 or greater SDS in 20% (Supplemental Figure 3).

In adulthood, IGF-1 concentrations tended to be high, being 0 or greater SDS in 87% and 2 or greater SDS in 20% of cases. On the contrary, pediatric IGF-1 concentrations were normal in all (Supplemental Figure 4A). Nine males (14%) had been diagnosed with partial GHD during childhood or adolescence. Seven of these are adults now, and six were retested after they reached adult height. The patient who was not retested in adulthood showed normal serum IGF-1 concentrations after cessation of recombinant human (rh) GH therapy. The six who were retested showed normal GH responses in all but one patient, in whom a peak GH of 1.24 ng/mL (reference range >5 ng/mL [6]) was observed during an insulin tolerance test at age 16 years. He continued rhGH therapy and is now 19 years old (see Supplemental Methods for a description of his mutation, p.Val985Ala). Two other adults were first tested for GHD at 79 and 66 years of age and showed normal GH responses during insulin tolerance test.

One patient did not show delayed bone age during childhood but rather had advanced bone ages (+1.9 y at ages 3.1 and 6.1 y) and tall stature (2.3 SD higher than target height SDS). At age 10 years, his testicular volume was 1.7 SDS for age, whereas no pubic hair, T, or dehydroepiandrosterone sulfate (DHEAS) production was observed. By that time, in the absence of a growth spurt, bone age was just 0.4 years above chronological age, and he was obese (BMI 2.3 SDS). He was treated for central hypothyroidism from birth and IGF-1 was always around −1.0 SDS.

### Puberty and gonadal function

GnRH testing was performed in nine pediatric patients aged 0.3–13.5 years (Supplemental Table 2). One infant, tested at 3 months of age, showed an LH peak of 37.5 U/L and FSH peak of 22.8 U/L, consistent with the physiological minipuberty in infants (7). A boy of 6.6 years old showed enlarged testes (3.4 SDS) and a borderline positive GnRH test (LH peak 5.2 U/L, Immulite immunoassay), but no other signs of pubertal development were observed in the years thereafter. Hormonal parameters were still prepubertal at 8 years of age (nonstimulated LH 0.3 U/L, FSH 4.3 U/L, T <0.7 nmol/L). Five patients aged between 7.3 and 11.6 years showed no activation of the hypothalamo-pituitary-gonadal axis despite having enlarged (2.8–4.4 SDS) or normal testes for age (0.2–0.5 SDS) at palpation. Lastly, the first evidently positive GnRH tests were observed in two patients at 12.7 years (LH peak 9.4 U/L) and 13.5 years (LH peak 8.3 U/L, testes >25 mL). In all these patients, including those with pubertal GnRH tests, pubic hair and T production (if these data were available) were absent, whereas testicular enlargement (≥4 mL) was observed in two of them as early as 2.6 years and 2.8 years. In most pediatric patients, ultrasonographic testicular volume was in the upper half of the reference range, but in 87% of adults it was 2.0 or greater SDS (Supplemental Figure 5). No testicular pathology was observed during ultrasonographic examinations, but two patients had suffered from testicular torsion at the age of 4 years and 74 years.

**Figure 1.**
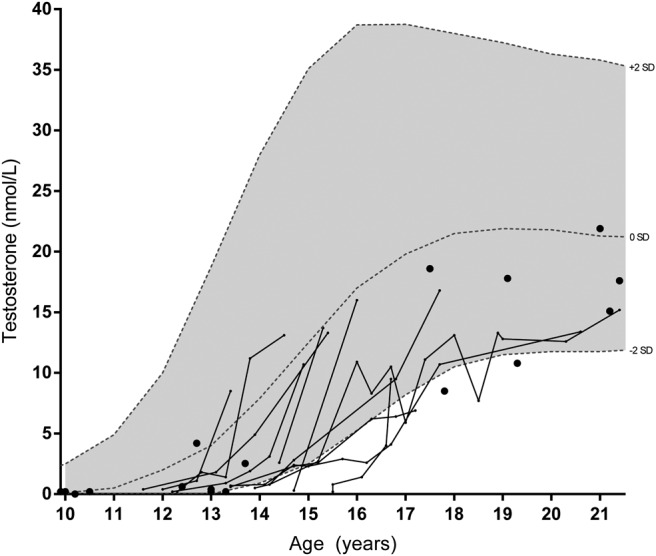
T concentrations in male patients. Lines represent longitudinal data and dots individual patients. Reference intervals were derived from Andersson et al (41).

Figure 1 displays (longitudinal) pediatric T values from the entire cohort, showing a late start of T rise in most. The GnRH test in 13 late-pubertal or adult patients (range 16.7–66.4 y) showed normal mean LH peaks of 26.3 U/L (SD 8.1 U/L) and mean FSH peaks of 15.5 U/L (SD 7.6 U/L). Mean T concentrations in adults were below the reference range median in 90% of adult patients, as was LH in 93% (Table 2). FSH was normal but relatively high compared with the low LH concentrations, resulting in an increased FSH to LH ratio. Inhibin B concentrations were normal, but the inhibin B to FSH ratio was below the reference range median in all adult patients (and decreased in 20%). Inhibin B concentrations in adults (n = 20) were not significantly correlated to ultrasonographic testicular volume (*P* = .677), and its correlations with FSH (*P* = .060) and T (*P* = .057) were almost significant.

**Table 2.** Adrenal and Gonadal Functioning in Male Adults

	n	Values	Reference Range
Age, y	32	30.9 (23.4–66.3)	
LH, U/L	29	3.3 ± 1.5^a^	2.0–9.0
FSH, U/L	29	8.2 ± 6.5	1.5–12.5
FSH to LH ratio	29	2.2 (1.8–2.7)^a^	0.7–1.2
T, nmol/L	31	14.0 ± 5.0^a^	8.0–31.0
SHBG, nmol/L	25	29.6 ± 15.5^a^	20–55
Inhibin B, ng/L	20	253.6 ± 111.0	150–400
Inhibin B to FSH ratio	20	31.5 (17.2–55.6)^a^	15–303
AMH, μg/L	21	7.0 (2.7–13.0)	2.0–14.0
Androstenedione, nmol/L	24	6.3 (3.4–9.0)	2.0–10.0
DHEA, nmol/L	20	9.7 (5.7–19.3)^a^	7.0–60.0
DHEAS, μmol/L	22	3.3 (1.3–4.9)^a^	2.0–15.0

Abbreviation: AMH, anti-Müllerian hormone. Data are presented as median (interquartile range) or mean ± SD.

a Different from population median at *P* < .05.

Two patients had been treated with T during puberty (1 and 3 years). Fertility was preserved in all evaluable individuals, except for one patient who was unable to reproduce and was diagnosed with azoospermia and was resistant to treatment with FSH and LH.

### Adrenal function

**Figure 2.**
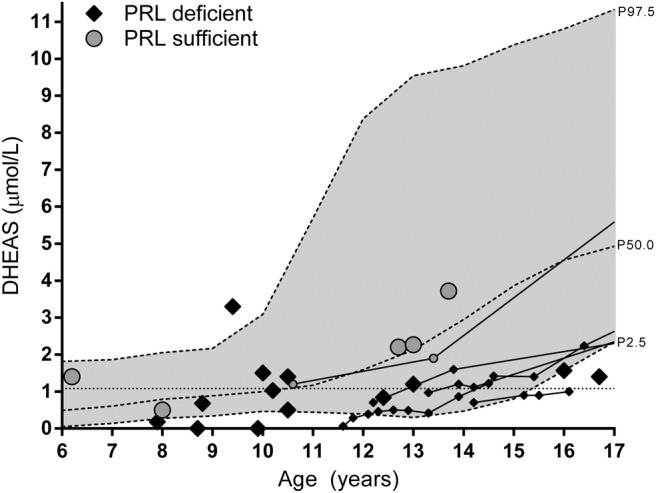
DHEAS concentrations in male patients around the age of biochemical adrenarche (1.084 μmol/L, dotted line). Lines represent longitudinal data, and the larger diamonds/dots are data from individual patients. Smoothed reference intervals were derived from Elmlinger et al (42). PRL, prolactin.

Figure 2 shows pediatric DHEAS plasma concentrations, revealing lower concentrations in those with prolactin deficiency (β = −1.357 nmol/L, *P* = .001, corrected for age). Longitudinal data on DHEAS were available in four patients with prolactin deficiency, showing a late biochemical adrenarche (DHEAS >1.084 μmol/L [8]) at 13–16 years. Adrenal steroids from 24-hour urine were collected in five patients aged 8.7, 8.8, 10.1, 12.4, and 13.0 years. Androgen metabolites (17-ketosteroids) were close to the lower limit of the reference range in all patients (Supplemental Figure 6).

In adults, dehydroepiandrosterone (DHEA) and DHEAS were below the reference range median in nearly all and decreased in 40% and 18%, respectively (Table 2). Androstenedione was generally normal, and no association between prolactin deficiency and adult adrenal steroid values was observed.

Cortisol concentrations were normal in all evaluated adult patients, and neither signs nor symptoms of hypocortisolism were observed. Hydrocortisone replacement was prescribed to only one 75-year-old patient, after showing an insufficient cortisol rise after administration of CRH (0.410 μmol/L, reference range >0.550 μmol/L) but a normal response to synthetic ACTH (0.840 μmol/L). Six of the 28 patients whose adrenal axis was evaluated shortly after birth (21%) were diagnosed with hypocortisolism because of low random cortisol values or abnormal low-dose ACTH testing results. However, hydrocortisone replacement could be stopped within a few years in all, after the finding of normal random cortisol values or adequate cortisol responses to synthetic ACTH (0.610–0.700 μmol/L).

### Metabolic parameters

Sixty-seven percent of male children were classified as overweight, and 21% as obese. Compared with the general population, BMI was greater than 2.0 SDS in 37%. Waist circumference was increased (>2.0 SDS) in 57%, and fat percentage in 29% (Supplemental Table 3). Lipids were generally normal, although three children aged 0.8–3.5 years showed increased total and LDL-cholesterol (cholesterol 2.7–3.6 SDS, LDL 2.7–4.5 SDS). All three were obese (BMI 2.2–3.1 SDS), and two were treated with levothyroxine. The untreated boy (0.8 y old) also showed increased triglyceride concentrations (4.03 mmol/L, reference range 0.21–2.01 mmol/L), which improved after the start of levothyroxine replacement at 1.3 years (1.22 mmol/L). BMI-corrected leptin concentrations were slightly increased because 84% showed concentrations of 0.0 or greater SDS and 21% 2.0 or greater SDS. Fasting concentrations of glucose, insulin, and C-peptide were generally normal.

Increased BMI (>25 kg/m^2^) was observed in 73% of adult males (general Dutch population: 53.8%, *P* = .017 [9]), but obesity (BMI > 30.0 kg/m^2^) was observed in only 17% (general population: 14.1%, *P* = .623). Furthermore, waist circumference was increased (≥94.0 cm) in 60% and fat percentage in 20% (Supplemental Table 3). Lipid concentrations were generally normal, as were BMI-corrected leptin concentrations, glucose, and insulin, but C-peptide was increased in 40%. FT_4_ concentrations were not significantly associated with any of these parameters (average *P* = .509). Hypertension was diagnosed and treated in four patients (13%), type 2 diabetes mellitus in three (10%), and dyslipidemia in four (13%) (all >60.0 y).

### Brain

Four patients were diagnosed with attention-deficit disorder, one with attention-deficit hyperactivity disorder, one with social-deficit hyperactivity disorder (10), and one showed deficits in attention and concentration at neuropsychological testing (13 y old). All of these patients were younger than 25 years, and four had been treated with psychostimulants. One other patient was diagnosed with a pervasive developmental disorder, not otherwise specified. In 8 of 24 available magnetic resonance imaging scans (six neonates), variable degrees of widening of the cerebrospinal fluid containing spaces were observed. The widening was located at the lateral and third ventricle in one but in the others mostly in the peripheral spaces: around the cerebellum in one, frontal in three, and in the basal cistern in three. In one of these six patients, however, a frontoparietal hygroma was diagnosed and treated with a ventriculoperitoneal shunt after being evaluated for macrocephaly at 6 months old (head circumference 5.1 SDS). In the remaining 14 patients, neuroimaging showed no abnormalities, other than hypoplasia of the corpus callosum in one case. No signs of cognitive impairment were observed.

### Female carriers

In female heterozygous carriers, FT_4_ was below the lower limit in 18% and in the lower tertile of the reference range in 60% (Supplemental Figure 1B). IGF-1 concentrations were 2.0 or greater SDS in 31% (mean ± SD: 1.1 ± 1.7, n = 42, Supplemental Figure 4B), and mild prolactin deficiency was present in 22%, although no one reported a history of problems with lactation. TRH tests were performed in six adult females (three of whom had decreased basal FT_4_), showing normal or exaggerated TSH peaks at 20 minutes (8.0–32.4 mU/L; reference range >2.8 mU/L). Five of 10 female carriers with decreased FT_4_ started treatment with levothyroxine after initial investigations, and all reported improvement of energy levels. Eight adult females underwent thyroid ultrasonography, showing a thyroid volume below the reference range median (<10.0 mL) in seven (two were on levothyroxine replacement) and less than the 2.5th percentile (<4.8 mL) in two (untreated). No evident relation with X-chromosome inactivation was observed because those with skewed inactivation (5 of 29 informative test results) showed central hypothyroidism in only two without hypoprolactinemia. Age at menarche was delayed (>14.5 y [11]) in 15 of 48 females (31%) with available data. No correlation was found between FT_4_ concentrations and age at menarche, and no fertility issues were reported. Cortisol was normal in all females (0.372 ± 0.202 μmol/L; reference range 0.100–0.600 μmol/L).

Females showed a metabolic profile similar to males, with increased BMI in 55%, waist circumference in 57%, and fat percentage in 36% (Supplemental Table 3). Triglycerides, cholesterol, high-density lipoprotein (HDL), and BMI-corrected leptin were generally normal, but high-density lipoprotein (LDL) was increased in 37%. Glucose, insulin, and C-peptide were generally normal. FT_4_ concentrations were significantly correlated to waist circumference (r = 0.345, *P* = .022), triglyceride concentrations (r = 0.375, *P* = .010), and fasting glucose (r = 0.409, *P* = .005). One adult female was treated for hypercholesterolemia (63.9 y), one for hypertension (62.2 y), and none for diabetes mellitus.

For all characteristics of IGSF1 deficiency in males and females, no clear genotype-phenotype relationship was observed, and signs and symptoms varied within families with the same mutation.

## Discussion

The results of this case series of patients with the X-linked IGSF1 deficiency syndrome, the largest to date, reveal new symptoms and expand the information on previously reported symptoms. Despite having been discovered only recently, this syndrome already encompasses more unique mutations and patients than all other known genetic causes of isolated central hypothyroidism combined (*TSHB* [12–16] and *TRHR* [17, 18]). An overview of the clinical features of IGSF1 deficiency is presented in Table 3, and recommendations for genetic evaluation and clinical management are provided in Table 4.

**Table 3.** Clinical Features of the X-Linked IGSF1 Deficiency Syndrome

Features	Values, %
Hemizygous males	
Central hypothyroidism	100
Low-normal T concentrations in adulthood	88
Adult macroorchidism	88
Delayed pubertal T rise, early/normal timing of testicular growth	75^a^
Mild problems with attentional control	75^a^
Small thyroid gland	74
Increased waist circumference in adults	59
Prolactin deficiency	61
Late biochemical adrenarche	50^a^
Increased waist circumference in children	57
Decreased DHEA in adulthood	40
Benign external hydrocephalus	33^a^
Increased birth weight	26
Hypocortisolism in infancy	21
Increased IGF-1 concentrations in adulthood	20
Increased head circumference	20
GHD in childhood	16
Heterozygous females	
Delayed age at menarche	31
Prolactin deficiency (non-symptomatic)	22
Central hypothyroidism	18

Based on reported data from current study and other reports (1, 3–5).

a Estimated based on limited data.

**Table 4.** Recommendations for Clinical Management of IGSF1 Deficiency

Indications for Mutational Analysis of *IGSF1*					
Central hypothyroidism of unknown cause, especially when accompanied by the following					
X-linked inheritance pattern					
Deficiency of prolactin or GH					
Disharmonious pubertal development, macroorchidism, or delayed adrenarche					
Screen appropriate family members after a mutation is discovered					
**Diagnosis and Follow-Up**	**Children**		**Transition**	**Adults**	
	**Diagnosis**	**Follow-Up**		**Diagnosis**	**Follow-Up**
Males					
History, physical examination	X^a^	X^a^	X	X^b^	X^b^
FT_4_, TSH	X	X^c^	X	X	X^c^
T	X	Annual if testes ≥4 mL	X	X	
Prolactin	X			X	
Dynamic adrenal axis testing	X^d^				
Cortisol (early morning)				X^e^	
IGF-1	X	^c^		X	
Cholesterol, TG, HDL, LDL, glucose	X	^c^	X	X	^c^
If growth failure or low IGF-1:					
(Primed) GH stimulation test, hand x-ray	X	X	^f^		
Females					
History, physical examination	X^a^			X^b^	
FT_4_, TSH	X	X^g^		X	^h^
Cholesterol, TG, HDL, LDL, glucose	X	^c^		X	^c^
Treatment					
Levothyroxine	In all male children. Trial course in male adults and in females with decreased FT_4_ or low-normal FT_4_ in combination with features suggestive of tissue hypothyroidism				
Hydrocortisone	In neonates with impaired cortisol response in low-dose ACTH test (<0.550 μmol/L); reevaluate after 1 y				
rhGH	In case of >1.0 SD deviation of growth, height <−2 SD or low growth velocity and impaired GH response in (primed) GH stimulation test				
T	In case of a delay in pubertal development in males (pubic hair stage 1 and/or prepubertal T at 14.0 y)				

Abbreviations: LT4, levothyroxine; TG, triglycerides.

a Height, weight, head circumference, pubic hair, testicular volume, heart rate. Periodic evaluation is done at the discretion of the treating physician.

b BMI, waist circumference, signs and symptoms of hypothyroidism and, if treated with levothyroxine, of hyperthyroidism. Periodic evaluation is done at the discretion of the treating physician.

c Follow-up at the discretion of the treating physician.

d Dynamic testing may be preceded by randomly measured plasma or serum cortisol concentrations. Sufficiently high concentrations make central adrenal insufficiency unlikely. However, low concentrations do not prove adrenal insufficiency. In case of a low random cortisol concentration in a neonate, we suggest performing a low-dose ACTH test. If abnormal (cortisol response <0.550 μmol/L), then treat with hydrocortisone. Reevaluate after 1 year.

e Sufficiently high concentrations make central adrenal insufficiency unlikely. A low concentration warrants dynamic adrenal axis testing.

f Repeat GH stimulation test at transition in patients treated for GH deficiency.

g Age 0–3 years, at least yearly and also if hypothyroidism is absent. Age 4–18 years: at discretion of treating physician.

h Preconception and during pregnancy. If hypothyroid, follow-up is done at the discretion of the treating physician.

### Central hypothyroidism and growth

Central hypothyroidism remains the key feature of IGSF1 deficiency, being present in all male cases. Because central hypothyroidism was previously also proposed to be the key feature (1, 2) and most new index cases were discovered based on this sign, its prevalence might be subject to selection bias. Nevertheless, all nonindex cases also showed central hypothyroidism. Growth velocity was usually decreased and bone age delayed (2), and partial GHD was diagnosed in 14%. We therefore advise stringent follow-up of growth, supplemented by IGF-1 concentrations, bone age determination, and (primed) GH stimulation tests in case of growth failure. Because GHD proved transient in most, but not all, patients treated with rhGH, GH stimulation tests should be repeated after reaching adult height.

### Pubertal development and adult gonadal functioning

The pubertal rise in T was often delayed, in contrast to a normal or even advanced start of testicular growth. The mechanism of this disharmonious pubertal development as well as the adult macroorchidism has not yet been elucidated. However, current data in the limited number of pubertal patients studied suggest a combination of both central and testicular causes. A central cause of the delayed T rise is supported by slightly delayed activation of the hypothalamic-pituitary-gonadal axis in GnRH stimulation tests, with relatively high FSH values that could contribute to testicular enlargement (15, 16). Additionally, testicular function might be altered through decreased penetrance of thyroid hormone in the testis, resulting in early and prolonged proliferation of Sertoli cells (causing macroorchidism) and decreased receptivity of Leydig cells to LH (causing delayed T rise) (15, 16). The normal inhibin B concentrations (a marker of Sertoli cell number and stimulated by FSH) despite macroorchidism and relatively high FSH concentrations might indicate decreased Sertoli cell function or decreased negative feedback of inhibin B at the pituitary level, although previous investigations reported no evidence for the latter (19). We advise to monitor pubertal development annually using height, pubic hair staging, testicular volume, and T concentrations (when testes reach ≥4 mL). Treatment with T should be considered when pubic hair stage 1 and/or prepubertal T concentrations are observed at the age of 14.0 years or greater. We discourage screening for *IGSF1* mutations in patients with delayed puberty and a normal thyroid function (20).

### Neonatal hypocortisolism

Although hypocortisolism was diagnosed in 21% of newborns (available data in 28 males [41%]), this condition proved transient within a few years in all. In some newborns, hypocortisolism was based on only randomly measured cortisol concentrations, which may have resulted in overestimation of its prevalence. Because of these two points, plus the absence of IGSF1 expression in corticotroph cells of the rat pituitary gland (21), we consider the chance that the adrenal axis is perturbed in IGSF1 deficiency remote. Nevertheless, we cannot rule out true neonatal hypocortisolism, and given the potentially dangerous nature of this condition we suggest dynamic adrenal axis testing in all newborns with central hypothyroidism. If adrenal insufficiency is diagnosed, hydrocortisone treatment should be initiated before the start of levothyroxine replacement because of the risk of initiating symptomatic adrenal insufficiency (22). Consequently, if dynamic testing cannot be performed, prophylactic glucocorticoid replacement should be considered just like in other causes of central hypothyroidism (23).

### Brain

Variable degrees of widening of the liquor containing spaces were observed in 8 of 24 magnetic resonance imaging scans. However, most findings were in neonates in whom this can be a variation of normal development known as benign external hydrocephalus (BEH) (24). BEH is the most prevalent cause of (familial) macrocephaly and usually resolves by the age of 2 years, although macrocephaly may persist. Increased head circumference was indeed observed in 20% of patients, and has also been reported in other causes of congenital hypothyroidism (25) and in patients with resistance to thyroid hormone due to a mutation in the *THRA* gene (26). Although one patient was treated for frontoparietal hygroma, we do not believe that these results warrant standard cerebral imaging in patients with IGSF1 deficiency and a normal head circumference, given the low prevalence and benign character of BEH. In addition, we previously reported mild but consistent deficits in attentional control (27), and seven patients (10.1%) were diagnosed with disorders related to attention (pediatric population prevalence of ADHD: 7.2% [28]). Treating physicians should be aware of these potential problems. Also, problems with gross motor skills should be recognized early because four patients benefited from physical therapy.

### Additional symptoms

First, we observed (very) small thyroid glands, especially in patients with suppressed TSH. This finding likely results from decreased TSH signaling (29–33) and warrants caution when patients on long-term treatment consider stopping levothyroxine replacement and in pregnant heterozygous females. Second, we observed increased birth weight in one-third of patients, in accordance with two of six Japanese cases of IGSF1 deficiency (5) as well as with other causes of congenital hypothyroidism (34). Third, a late adrenarche was observed in patients with prolactin deficiency, although data were limited. Prolactin receptors are highly expressed in the adrenal gland and synergize with ACTH to augment adrenal androgen secretion (35–37). Furthermore, experimental lowering of prolactin concentrations decreases DHEAS, whereas DHEAS is elevated in hyperprolactinemia (38, 39). Prolactin deficiency may therefore be considered as the main cause of late adrenarche in IGSF1-deficient patients. An association between adrenal steroids and prolactin concentrations was absent in adults.

### Treatment

The degree of hypothyroidism varied, and for all patients it was unknown to what extent FT_4_ concentrations were below individual and optimal set points and how long-term congenital hypothyroidism may have affected this set point. There are, however, several arguments in favor of treatment. We observed prolonged jaundice, growth delay, obesity, and sometimes dyslipidemia in untreated children who later responded well to the initiation of treatment (1). In male adults, BMI-corrected SHBG concentrations (a marker of liver hypothyroidism) were significantly correlated to FT_4_ concentrations, implying suboptimal thyroid hormone exposure in untreated individuals. We also observed higher waist circumference, triglycerides, and glucose in females with lower FT_4_ concentrations. Male and female adults who started treatment after diagnosis reported major improvements in energy levels, as previously described in other hidden forms of central hypothyroidism (23), although long-term results in our patients are unavailable. On the other hand, untreated adults were generally well functioning, well educated, of normal height, rarely showed dyslipidemia or cardiovascular disease, and were not more obese than their treated peers. Nevertheless, we advise treatment of all children with levothyroxine, commencement of a trial course in all male adults and in female adults with low (-normal) thyroxine concentrations, and reassessment of FT_4_ concentrations in females before and during pregnancy.

### Genetic evaluation

We advise genetic evaluation of all patients with central hypothyroidism of unknown cause for *IGSF1* mutations, especially when accompanied by an X-linked inheritance pattern, prolactin or GH deficiency, disharmonious pubertal development, macroorchidism, or delayed adrenarche. Because asymptomatic adult carriers are likely to benefit from treatment with levothyroxine, family members should be evaluated based on the X-linked inheritance pattern. All children of female carriers (and female children of male patients) should be screened at birth with TSH and T_4_.

In conclusion, this study describes the phenotype of the X-linked IGSF1 deficiency syndrome in the largest cohort known to date. The results include new symptoms, as well as an expansion of the information on known symptoms, allowing us to formulate recommendations for clinical management.
